# Enhanced laccase-mediated transformation of diclofenac and flufenamic acid in the presence of bisphenol A and testing of an enzymatic membrane reactor

**DOI:** 10.1186/s13568-018-0546-y

**Published:** 2018-02-24

**Authors:** Veronika Hahn, Mareike Meister, Stephan Hussy, Arno Cordes, Günther Enderle, Akuma Saningong, Frieder Schauer

**Affiliations:** 1 Institute of Microbiology, Ernst-Moritz-Arndt-University Greifswald, Friedrich-Ludwig-Jahn-Str. 15, 17487 Greifswald, Germany; 2 Leibniz Institute for Plasma Science and Technology (INP Greifswald e.V.), Felix-Hausdorff-Str. 2, 17489 Greifswald, Germany; 3 Atec Automatisierungstechnik GmbH, Emmi-Noether-Str. 6, 89231 Neu-Ulm, Germany; 4 ASA Spezialenzyme GmbH, Am Exer 19 C, 38302 Wolfenbüttel, Germany; 5 EurA Consult AG, Max-Eyth-Str. 2, 73479 Ellwangen, Germany

**Keywords:** Quinone, Bioremediation, Detoxification, Pharmaceuticals, Biodegradation, *Vibrio fischeri*

## Abstract

The inadequate removal of pharmaceuticals and other micropollutants in municipal wastewater treatment plants, as evidenced by their detection of these substances in the aquatic environment has led to the need for sustainable remediation strategies. Laccases possess a number of advantages including a broad substrate spectrum. To identify promoting or inhibitory effects of reaction partners in the remediation processes we tested not only single compounds—as has been described in most studies—but also mixtures of pollutants. The reaction of diclofenac (DCF) and flufenamic acid (FA), mediated by *Trametes versicolor* laccase resulted in the formation of products, which were more hydrophilic than the respective reactant (reactant concentration of 0.1 mM; laccase activity 0.5 U/ml). Analyses (HPLC, LC/MS) showed that the product **1a** and **1b** for DCF and FA, respectively, to be a *para*-benzoquinone imine derivative. The formation of **1a** was enhanced by the addition of bisphenol A (BPA). After 6 days 97% more product was formed in the mixture of DCF and BPA compared with DCF tested alone. Product **1a** was also detected in experiments with micropollutant-supplemented secondary effluent. Within 24 h 67% and 100% of DCF and BPA were transformed, respectively (25 U/ml). Experiments with a membrane reactor (volume 10 l; phosphate buffer, pH 7) were in good agreement with the results of the laboratory scale experiments (50 ml). EC_50_-values were also determined. The data support the use of laccases for the removal or detoxification of recalcitrant pollutants. Thus, the enzyme laccase may be a component of an additional environmentally friendly process for the treatment stage of wastewater remediation.

**Electronic supplementary material:**

The online version of this article (10.1186/s13568-018-0546-y) contains supplementary material, which is available to authorized users.

## Introduction

The daily use of anti-inflammatory drugs such as diclofenac (DCF) or flufenamic acid (FA) in creams or tablets, as well as of plastic materials which release bisphenol A (BPA), contributes to pollution with these environmentally dangerous compounds. BPA is used in plastic industries; consequently the release from different sources is conceivable such as packages for food and drink, varnishes, paintings and glues (Barnabe et al. 2009). The main entries for pharmaceuticals such as DCF or FA in ground and surface water are sludge or effluents of wastewater treatment plants (WWTP) where the pollutants are insufficiently degraded. Another source is dung and manure of farm animals which were treated with these substances (Ternes 1998; Deblonde et al. 2011; Samaras et al. 2013; Carmona et al. 2014).

Only 17–69% of DCF is removed in sewage treatment plants (Ternes 1998; Zwiener et al. 2000; Heberer 2002a; Heberer and Feldmann 2005; Deblonde et al. 2011; Samaras et al. 2013). FA is also insufficiently removed in WWTP. Moreover, Gracia-Lor et al. (2012) and Carmona et al. (2014) described for DCF and FA higher concentrations in the effluent than in the corresponding influent of spanish WWTP. The authors proposed a release as result of a cleavage of transformation products, deconjugation of metabolites and desorption from organic material.

DCF, FA and BPA were found repeatedly in surface water. Ternes (1998) reported for DCF a median of 0.15 µg/l and a maximum of 1.20 µg/l for German river and stream water. In Berlin, up to 0.5–1 µg/l and 0.38 µg/l were found in the Teltowkanal and in ground water, respectively (Heberer 2002b). FA was detected in river and tap water with average concentrations of 21 and 16 ng/l, respectively (Carmona et al. 2014). BPA was also found in surface and ground water (Loos et al. 2009, 2010). In small streams of the German region called Hessisches Ried BPA concentrations of up to 1.92 µg/l were detected (Quednow and Püttmann 2008).

DCF, FA as well as BPA may cause toxic effects. In rainbow trout DCF accumulates in the bile to a factor of approximately 580, and additionally the tested environmentally relevant concentrations caused necrosis in the kidney (Mehinto et al. 2010). The endocrine disrupting compound BPA leads to the induction of feminization in aquatic organisms, such as frogs (Levy et al. 2004; Bhandari et al. 2015). Because of the potential risk for human health, DCF was included in a watch list of emerging pollutants by the EU (Commission 2013, 2015). Nadanaciva et al. (2013) working with zebrafish, showed a high toxicity for FA, but it was not possible to locate the morphological changes because the difference between the FA concentration with no effect where no death occur (NOEC) and the concentration causing 100% lethality (LC_100_) was too small. Thus, for FA as well as DCF the same NOEC of 10 µM was determined whereas the LC_100_ was 30 µM for FA and 300 µM for DCF, allowing the determination of morphological changes, such as liver browning, only for DCF (Nadanaciva et al. 2013). Furthermore, in vitro test showed a considerable effect of FA on liver mitochondria (Nadanaciva et al. 2013).

The repeated detection of pharmaceutical residues in the aquatic environment demonstrates the urgent need for efficient wastewater treatment processes. In this regard, the enzyme laccase has been considered as a tool for a sustainable remediation strategy and for additional purification steps in wastewater treatment. Laccases [E.C. 1.10.3.2] can oxidize a broad range of compounds such as phenols or amines (Keilin and Mann 1939; Bollag et al. 1988; Thurston 1994; Mikolasch and Schauer 2009) by one-electron-reactions (Nakamura 1960; Solomon et al. 2001, 2008; Munk et al. 2017). In these laccase-mediated oxidations, radicals are formed which can undergo two possible reactions. The first involves binding to other compounds, while the second involves cleavage (Hahn et al. 2014). An important advantage of laccases, in particular for remediation purposes, is their need for atmospheric oxygen as the only co-substrate. Thus, oxygen is reduced to water.

Despite the well known removal of water contaminants such as BPA or other phenols, DCF and estrogens by free or immobilized laccase of *Trametes versicolor* (Auriol et al. 2008; Catapane et al. 2013; Ammann et al. 2014) has until now in most studies been tested only single compounds. The transformation in mixtures of micropollutants was described in most cases without the distinction of the influencing effect of the single compounds on the degradation in these mixtures (Tran et al. 2010; Nguyen et al. 2014; Asif et al. 2017b). To determine the effect of multiple contaminants on transformation rate and product formation we employed mixtures of pollutants. In addition, removal of FA was tested due to limited data.

The laccase of *T. versicolor* was employed for the transformation of DCF, FA and BPA and combinations of the substances were also tested. The removal of these test substances as well as product formation was determined by HPLC analyses. The structural characterization of products was performed by LC/MS.

The results of this study will be used for the development of an enzyme-based membrane reactor designated as an additional or tertiary treatment stage in sewage treatment plants.

## Materials and methods

### Chemicals

All chemicals used were of analytical grade and were used as received.

Diclofenac sodium salt, flufenamic acid, bisphenol A, 4′-hydroxydiclofenac and ABTS (2,2′-azino-bis(3-ethylbenzothiazoline-6-sulfonic acid) diammonium salt) were purchased from Sigma-Aldrich Chemie GmbH (Steinheim, Germany). 5-Hydroxydiclofenac was obtained from Toronto Research Chemicals (Toronto, Canada). Potassium dihydrogen phosphate and di-sodium hydrogen phosphate dihydrate were purchased from Carl-Roth GmbH and Co. KG (Karlsruhe, Germany).

### Enzyme

The laccase from *T. versicolor* was obtained from ASA Spezialenzyme GmbH (Wolfenbüttel, Germany). The company markets the enzyme under the name “laccase C”. It was produced by a fed-batch fermentation process. The fungal biomass was separated by centrifugation and the enzyme-containing supernatant was concentrated by ultrafiltration. After addition of stabilizing agents the retentate of the ultrafiltration step was lyophilized. The laccase is active within pH 3.0–7.5 (pH optimum of 5). It was used as received (activity > 800 U/g; substrate:syringaldazine).

For transformation experiments the laccase was used in Sørensen’s phosphate buffer at pH 7 in accordance with the pH 7–7.5 of the secondary wastewater.

### Experimental procedures for transformation in phosphate buffer or secondary effluent

For the transformation assays the respective compounds (initial reactant concentration: 0.1 mM) were incubated in a final volume of 50 ml in 500-ml flasks. The reaction solution was phosphate buffer or secondary effluent. The laccase activity was 0.5 U/ml for reactions in phosphate buffer and, additionally, 2.5 and 25 U/ml in wastewater. The secondary effluent from a wastewater treatment plant (85,000 inhabitant equivalents Greifswald, Germany) was 0.22 µm filtered and spiked with the respective compound.

In controls the respective compounds were incubated in phosphate buffer or secondary effluent without laccase. Reaction mixtures were incubated with agitation at 150 rpm at 25 °C in the dark.

### Reactor setup and operation

**Fig. 1:**
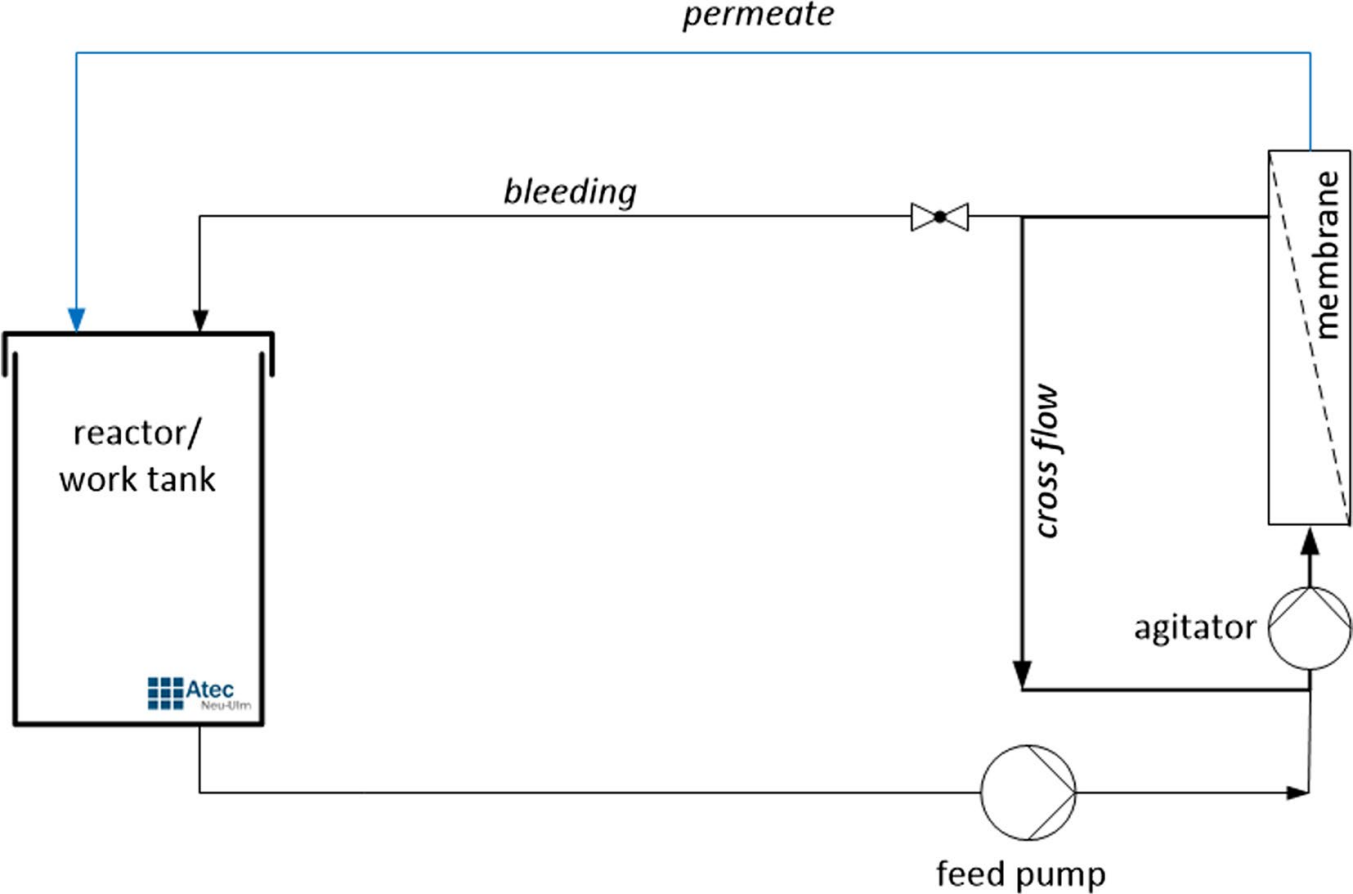
Scheme of the reactor equipped with an Atec ultrafiltration system

The laboratory reactor was equipped with an Atec ultrafiltration system to avoid loss of laccase (Fig. 1). From the different membranes tested for that purpose a tubular ceramic membrane element with a pore size of 10 nm gave the best results (Membrane: gamma-Al_2_O_3_, support: alpha Al_2_O_3_, manufacturer:Inopor). Like Atec’s commercial Miditube filtration systems the laboratory unit uses a unique design allowing almost independent control of cross flow over the membrane surface, and trans membrane pressure. Filtration with low trans membrane pressures leads only to the formation of thin layers on the ultrafiltration membranes extending the time interval to back flush.

Depending on the temperature (20–27 °C) the permeate flow of 26–36 l/(m^2^ h bar) was constant during experiment if working pressure was kept low (1.1 bar). The ratio of bleeding to permeate flow was adjusted to 15:1. System was run continuously during duration of the experiment (4 h).

The respective substances (initial reactant concentration: 0.1 mM) were tested in a final volume of 10 l in phosphate buffer. The laccase activity employed was 0.5 U/ml.

The decrease of reactants and the products formed were analyzed by HPLC.

### Measurement of laccase activity

The activity of laccase was determined spectrophotometrically at 420 nm with ABTS as substrate (Bourbonnais and Paice 1990) using the method described by Jonas et al. (1997) at pH 5. 1 U is defined as the turnover of 1 µmol/ml min

### Analytical HPLC (high-pressure liquid chromatography)

For routine analysis, the reaction mixtures were analyzed using an HPLC system LC-10AT VP (Shimadzu, Germany) consisting of a FCV-10AL VP pump, SPD-M10A VP diode array detector, and a SCL-10A VP control unit controlled by Class-VP version 6.12 SP5. Substances were separated on an endcapped, 5-µm, LiChroCART^®^ 125-4 RP18 column (Merck, Darmstadt, Germany) run at a flow rate of 1 ml/min. The solvent system used consisted of a gradient of methanol (eluent A) and 0.1% phosphoric acid (eluent B), starting from an initial ratio of 10% A and 90% B and reaching 100% methanol within 14 min. Elution with methanol was continued for a further 6 min.

### Structural characterization of products by LC/MS (liquid chromatography/mass spectrometry)

The reaction mixtures and isolated products were characterized using a LC/MS system. The atmospheric pressure ionization (API) mass spectrometry experiments were performed on an Agilent Series 1200 HPLC system with diode array detector and an Agilent 6120 quadrupole mass spectrometer (Waldbronn, Germany). The MS was run with the electrospray ionization (API-ES) source in positive mode (dry and nebulizer gas: nitrogen; nebulizer pressure: 45 psig; drying gas flow: 10 l/min; drying gas temperature: 350 °C; capillary voltage: 4 kV; fragmentor voltage: 75 V). HPLC separation was performed on a Zorbrax SB-C18 (2.1 × 50 mm, 1.8 µm) column (Agilent, Waldbronn, Germany), at a flow rate of 0.07 ml/min. The solvent system consisted of a gradient of acetonitrile (eluent A) and 0.1% aqueous ammonium formate (eluent B), starting from an initial ratio of 10% A and 90% B and reaching 100% methanol within 7 min. Elution with methanol was continued for a further 6 min.

Experimental methods; UV–vis data and MS spectra for products **1a**_**I–III**_, **2**, and **1b**_**I,II**_ (Additional file 1: Table S1–S6) are available in the electronic additional file.

### Determination of toxicity using *Vibrio fischeri* (DIN EN ISO 11348, (DIN 38412-L34, DIN 38412-L341))

This marine bacterium is bioluminescent and emits light with a wavelength of 490 nm. The bioluminescence is reduced in the presence of toxic agents.

Samples were taken before and after laccase treatment from a membrane reactor (initial concentration of reactants: 0.1 mM, volume: 10 l; phosphate buffer; incubation time: 4 h).

For the determination of EC_20_ and EC_50_ the samples were inoculated with *Vibrio fischeri* (LCK 482, Hach Lange GmbH, Düsseldorf, Germany) and incubated for 30 min. The reduction of bioluminescence was measured photometrically (LUMIStox 300-Version 3.03 Hach Lange GmbH, Düsseldorf, Germany). The results were compared with a control containing physiological saline solution instead of the sample.

## Results

### Transformation of DCF, FA and BPA including structural characterization of the products formed

**Fig. 2:**
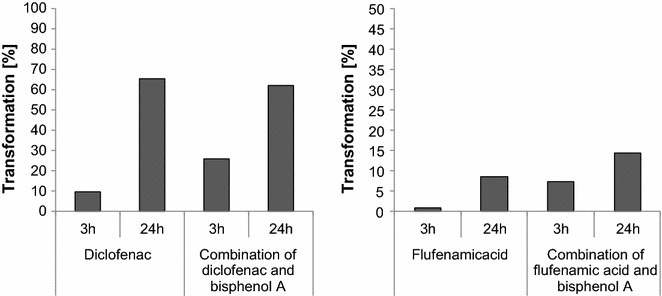
Laccase-mediated transformation [%] of DCF or FA treated individually or in combination with BPA in phosphate buffer (concentration of reactants 0.1 mM)

DCF, FA and BPA were transformed by laccase of *T. versicolor* to varying extents (concentration of reactants: 0.1 mM; Fig. 2). The reactant BPA was completely transformed within 24 h in the laccase-mediated reactions irrespective of whether it was tested individually or in combination with DCF or FA. The influence of added BPA on the transformation of the other two compounds was significant within 3 h. In the reaction with BPA the acids DCF and FA were 2.7 and 7 times faster transformed in the presence of BPA. After 24 h DCF transformation reached approximately 65% in both reactions whereas FA reached 9% (without BPA) and 15% (with BPA).

For all reactions different low- and high-molecular weight products were detected but due to the small quantities and difficult analyses (at least for the high-molecular weight products) only a selection of products were structurally characterized.

The laccase-mediated reaction of DCF leads to the formation of product **1a**_**I**_ which was detected after an incubation period of 6 days. In combination of DCF and BPA the product (**1a**_II_) was detected already after 3 h. Thus, within 6 days only 3% of **1a**_**I**_ was formed in the reaction of DCF (**1a**_**I**_: 0.39 µg/ml; 0.0013 mM) compared to the mixture of DCF and BPA (**1a**_**II**_: 13.34 µg/ml; 0.0430 mM).

The product **1a**_**I**_ was more hydrophilic (R_f_ 12.23 min) than DCF (R_f_ 14.18 min) and showed UV–vis absorption maxima at 202, 267 and 462 nm (Table 1). The LC/MS (API-ES positive mode) analyses of **1a**_**I**_ resulted in the detection of [M + H]^+^ ion multiplets at *m/z* (rel. intensity) 310.0, 312.0 and 313.8 (90:74:5; Additional file 1: Table S1) implying an hydroxylation and oxidation of DCF.

**Table 1:**
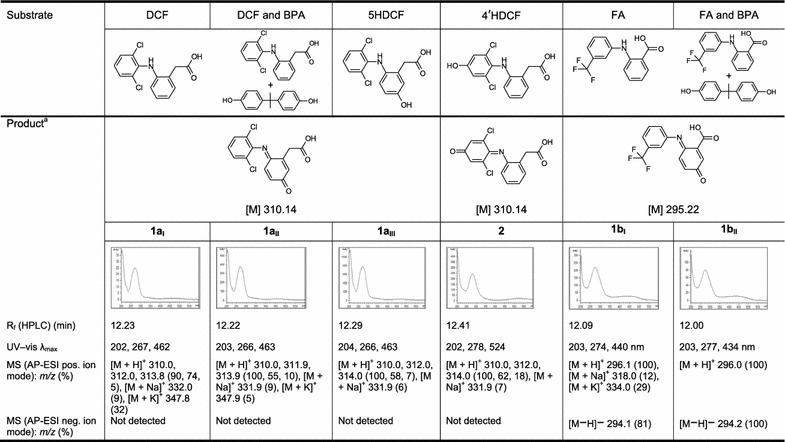
Structural data of products **1a**_**I–III**_ formed during laccase-mediated reactions of DCF alone or in combination with BPA as well as 5HDCF; data for product **2** from the reaction of 4′HDCF and for products **1b**_**I,II**_ from the reaction of FA alone or in combination with BPA are provided

According to the HPLC and LC/MS data products **1a**_**I**–**III**_ and **1b**_**I,II**_ have the same chemical structure, respectively

^a^Only Products of DCF and FA are shown

The UV–vis absorption maximum at 267 nm as well as the ion multiplets at *m/z* 310.0, 312.0 and 314.0 (Additional file 1: Table S3) were reported by Shen et al. (1999) for the *p*-benzoquinone imine derivative (**1a**_**III**_) of 5–hydroxydiclofenac (5HDCF) formed during incubation of DCF with liver microsomes. Thus, the reactions of DCF (alone or in a mixture with BPA) or 5HDCF resulted in the same product **1a**. For the laccase-mediated DCF transformation a hydroxylation at C5 with subsequent formation of a *p*-benzoquinone imine **1a**_**I**,**II**_ was presumed.

The 4′-hydroxydiclofenac (4′HDCF) was incubated with laccase to exclude the 4′-position for the hydroxylation. The reaction resulted in the formation of the corresponding quinone imine (**2**). The LC/MS (API-ES positive mode) analyses of **2** resulted also in the detection of [M + H]^+^ ion multiplets at *m/z* (rel. intensity) 310.0, 312.0, 314.0 (100, 62, 18; Table 1; Additional file 1: Table S4) but the retention time of 12.41 min as well as the UV–vis absorption maxima at 202, 278, 524 nm were different to those of **1a**_**I-III**_.

Similar to the reaction of DCF the reaction of FA alone and in combination with BPA resulted after 10 days in a more hydrophilic product (**1b**_**I**_ R_f_ 12.09 min or **1b**_**II**_ R_f_ 12.00 min) than FA (R_f_ 14.99 min). The LC/MS analyses (API-ES positive and negative mode) for the laccase-mediated reaction of FA resulted in the detection of [M + H]^+^ (rel. intensity) 296.1 (100), [M + Na]^+^ 318.0, [M + K]^+^ 334.0 and [M − H]^−^ 294.1 (Additional file 1: Table S5). The C4-position for the hydroxylation was assumed because of the similarities with the reaction of DCF, though no authentic standard was available. The amount of products (**1b**_**I**,**II**_) formed was too small to determine a dependency between product yield and BPA addition. The concentration of BPA may not have been high enough to promote the formation of **1b**_**II**_.

### DCF transformation alone and in combination with BPA in a membrane reactor and determination of toxicity

Experiments with a membrane reactor (volume of 10 l) were carried out to provide information about the efficiency of laccase-mediated transformation of pollutants.

**Fig. 3:**
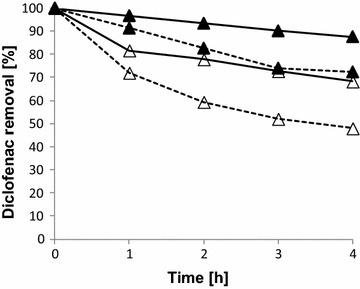
Transformation of DCF tested individually (black lines) and in mixture with BPA (dashed lines) in 500-ml flasks (laboratory scale; reaction volume: 50 ml; filled triangles) and in filtrate of a membrane reactor (reaction volume: 10 l; open triangle) in phosphate buffer (pH 7) and a laccase activity of 0.5 U/ml (concentration of reactants: 0.1 mM)

In samples of laboratory scale, and in the filtrate of a membrane reactor, DCF transformation was enhanced by the presence of BPA (Fig. 3). Thus, in both situations the removal of DCF was approximately 20% higher after 4 h in the mixture of DCF and BPA than without BPA. Furthermore, **1a**_**I**_ was not detected when DCF was tested alone due to the short incubation time of 4 h. In the combination of DCF and BPA the product **1a**_**II**_ was formed as in the 50 ml assay. The loss of laccase during the 4 h incubation in the reactor was negligible.

The reaction assays of the membrane reactor were tested for their toxicity against *Vibrio fischeri* before and after incubation with laccase (Table 2). In all tested assays the toxicity decreased with decreasing concentration of the pollutant/s caused by laccase-mediated transformation. After an incubation time of 4 h 32% of DCF was removed whereas in the mixture with BPA 52% of DCF was transformed. The reactions with BPA resulted in a complete transformation.

**Table 2:** Results of the toxicity test (test organism: *Vibrio fischeri*), including concentration of DCF and BPA, before and after laccase treatment in a membrane reactor (volume: 10 l; phosphate buffer; incubation time: 4 h)

Reaction	Concentration				Toxicity test (*Vibrio fischeri*)			
	DCF		BPA		EC_50_		EC_20_	
	mg/l	mM	mg/l	mM	mg/l	mM	mg/l	mM
DCF								
Before laccase treatment	31.81	0.10			11.36	0.0357	5.53	0.0174
After laccase treatment	21.70	0.07			19.50	0.0613	9.59	0.0302
BPA								
Before laccase treatment			22.83	0.10	5.24	0.0230	1.36	0.0060
After laccase treatment			0	0	22.16	0.0971	3.01	0.0132
DCF and BPA								
Before laccase treatment	31.81	0.10	22.83	0.10	3.91	0.0143	1.50	0.0055
After laccase treatment	15.27	0.05	0	0	12.41	0.0454	6.24	0.0225

For DCF and BPA the toxicity was reduced by 42 and 76%, respectively (according to EC_50_-values). In the reaction of DCF with BPA the toxicity was diminished by 69% after laccase treatment. The residual toxicity is probably caused by products formed or—in case of DCF—by the high concentration of reactant, which is left in the reaction assay. Nevertheless, the data show a clear decrease in toxicity due to laccase treatment. A longer incubation time would be expected to lead to higher transformation of DCF, which may cause a further decrease in toxicity.

### DCF transformation alone and in combination with BPA in municipal wastewater

A laccase activity of 0.5 or 2.5 U/ml was used for the experiments with the secondary effluent. However, this activity, as seen in the previous experiments, resulted in no transformation of DCF. 25 U/ml in wastewater was needed to achieve 67% transformation of DCF and complete removal of BPA within 24 h. In the mixture of DCF and BPA 33% and 65% were transformed, respectively.

Within 24 h **1a**_**I**_ was only detected in the reaction with DCF alone (**1a**_**I**_ 7.03 µg/ml; 0.0227 mM) in accordance with the faster transformation of DCF in this reaction compared to the reaction in a mixture with BPA.

## Discussion

Laccase-mediated reactions resulted in the transformation of micropollutants which are problematic for aquatic environment. The transformation efficiency of BPA, DCF, and FA mediated by *T. versicolor* laccase in the lab scale reactions (reaction volume 50 ml; phosphate buffer) was: BPA > DCF > FA. The laccase-mediated oxidation can be hindered by different factors such as steric problems through the size of the substrate-binding site (Tadesse et al. 2008; Galli et al. 2011).

Moreover, it is believed that laccase can only oxidize those substrates which have a redox potential below that of the enzyme (Xu et al. 2000). Thus, laccases were divided according to their redox potential into low (0.4–0.5 V, determined against a normal hydrogen electrode) and high redox potential (0.7–0.8 V) laccases (Xu 1996; Xu et al. 1998; Li et al. 1999; Frasconi et al. 2010). The laccase of *T. versicolor* belongs to the high redox potential laccases (0.785 V, Reinhammar 1972). The low redox potential of mono- and dihydroxylated aromatic compounds such as guaiacol or hydroquinone (0.5–0.6 V) allows an easy oxidation by laccase (Mai et al. 2001). The kind of substituents has also an influence on the oxidizability (Tadesse et al. 2008; Hahn et al. 2014). Thus, electron-withdrawing groups such as fluoro or chloro and carboxyl groups which are part of DCF and FA reduce the possibility for oxidation whereas electron-donating hydroxyl groups of BPA decrease the redox potential resulting in an easier oxidation.

The addition of BPA at least slightly enhanced the transformation of DCF and FA although complete transformation was not achieved. An incomplete biotransformation of DCF by laccase is not unexpected, and was also described for another laccase preparation of *T. versicolor* (Sigma-Aldrich Chemie GmbH, ref. 38429, Buchs, Switzerland) by Margot et al. (2013).

The reaction of DCF and BPA in 10 l phosphate buffer in the membrane reactor resulted also in enhanced transformation of DCF in the presence of BPA. This demonstrates that the scale up was successful. In both—the 50 ml and 10 l scale—experiments the removal of DCF was approximately 20% higher after 4 h in the mixture of DCF and BPA than without BPA.

Nair et al. (2013) described an efficient transformation of DCF by immobilized laccase of *Coriolopsis gallica* in a continuous stirred tank membrane reactor, though the reactor volume was only 50 ml (flow rate: 40 ml/h). Thus, 70% of the DCF was transformed within 80 h (initial concentration: 10 µM, laccase activity approximately 1 U/ml, McIlvaine buffer pH5). Nguyen et al. (2014) described the removal of BPA and DCF in an enzymatic membrane reactor using the laccase of *Myceliophthora thermophila*. Within 132 h 85% BPA and 60% DCF was continuously transformed (initial concentration 500 µg/l d, laccase activity approximately 90 µM/min, Milli-Q-water). A further experiment with the same laccase and a mixture of BPA, DCF with 28 other pharmaceutics such as naproxen or ibuprofen was described by Asif et al. (2017b). In the enzymatic reactor approximately 88% BPA and 45% DCF were transformed (initial concentration: 20 µg/l, laccase activity approximately 95–100 µM/min, Milli-Q-water).

The enhanced transformation of DCF and FA in the presence of BPA is striking. It suggests that BPA, or transformation products resulting from a cleavage of BPA, may act as mediators. Different authors described hydroxylated monoaromatic compounds such as 4-isopropenylphenol or 4-ethyl-2-methoxyphenol which were formed during laccase-mediated transformation of BPA (Fukuda et al. 2001; Chairin et al. 2013; Arca-Ramos et al. 2015). In a similar way, mediators can be formed in course of the degradation processes such as the depolymerization of lignin (or are produced by the fungus itself). Such mediators are small-molecular weight substances such as syringaldehyde or vanillin. These compounds may be oxidized by laccase and, in turn, can oxidize compounds which are not accessible for the laccase due to their high redox potential or to steric hindrance (Bourbonnais and Paice 1990; Baiocco et al. 2003; Wesenberg et al. 2003; Kunamneni et al. 2008; Tadesse et al. 2008; Mogharabi and Faramarzi 2014). Lloret et al. (2010) showed an improved transformation of DCF in the presence of different mediators such as syringaldehyde or 1-hydroxybenzotriazole.

### Formation of product **1a**

The formation of **1a** was promoted by the presence of BPA. Thus, only 3% of **1a**_**I**_ was detected after 6d in the reaction of DCF alone, compared to the reaction in the presence of BPA. In contrast, within the first 4 h of incubation, the transformation of DCF was approximately 20% higher in the reaction with BPA and nearly the same after 24 h. Thus, despite an only slightly enhanced transformation of DCF in a mixture with BPA the formation of **1a**_**I**,**II**_ is favored in the mixture suggesting an easier hydroxylation in the reaction with added BPA than in the reaction only with DCF.

In contrast with the reactions in phosphate buffer, **1a**_**I**,**II**_ was formed in the secondary effluent, in particular in the reaction with DCF alone, due to a slower removal of DCF in the mixture with BPA.

The product **1a**_**I**,**II**_ and **1b**_**I**,**II**_ is probably formed via a hydroxylation of DCF or FA by a nucleophilic attack of water on a laccase-generated cation radical, with subsequent oxidation resulting in a *para*-benzoquinone imine derivative. To the best of our knowledge, this is the first description of products **1a** and **1b** for laccase-catalyzed reactions. The laccase-mediated hydroxylation of *para*-dihydroxylated aromatic compounds was reported previously (Manda et al. 2007; Hahn et al. 2009) but interestingly the hydroxylation of DCF did not involve the well characterized substitution of a chloro group as previously described (Minard et al. 1981; Iimura et al. 1996; Kordon et al. 2010; Hahn et al. 2014). Hydroxylation on the chlorinated ring of DCF is probably hindered due to the electron withdrawing effects of the chloride atoms (Faber et al. 2012). The hydroxylation of DCF took place at the C5-position. Marco-Urrea et al. (2010) also described the formation of 5-hydroxydiclofenac in reactions with whole cells of *T. versicolor* cultures, whereas with isolated laccase only 4-(2,6-dichlorphenylamino)-1,3-benzenedimethanol was detected. In these laccase-mediated reactions again the C5 and additionally C3 was derivatized (Marco-Urrea et al. 2010).

The formation of the quinone imine derivative **1a** was also described for a reaction of DCF catalyzed by a peroxymonosulfate/Cobalt(II) system by a one-electron mechanism similar to laccase-mediated reactions (Ahmed et al. 2012). The C5-position for hydroxylation is favored both for the peroxymonosulfate/Cobalt(II) system, as well as for the electrochemical formation of the *para*-benzoquinone derivative (Ahmed et al. 2012; Faber et al. 2012).

DCF is metabolized by human cytochrome-P450 enzymes to a range of hydroxylated products, namely 4′-, 3′- and also 5-hydroxydiclofenac (so called phase I metabolites; Davies and Anderson 1997; Dorado et al. 2003). For humans, as well as fungi, the formation of 4′-hydroxydiclofenac is the primary transformation pathway of DCF, 3′- and 5-hydroxydiclofenac are only minor products (Davies and Anderson 1997; Webster et al. 1998; Dorado et al. 2003).

### Detoxification in a membrane reactor

The experimental data provided for the reaction course of DCF and BPA in a reaction volume of 50 ml and 10 l demonstrate that the laboratory scale results can be transferred to higher reaction volumes, which are necessary for biotechnological applications in membrane reactors.

The determined toxicity of DCF and BPA alone or in a mixture decreased after the treatment with laccase in the membrane reactor due to decreased concentrations of the reactants. Nevertheless it has to be bourne in mind, that the hydroxylated product 5HDCF (EC_50_ 39.59 mg/ml, 0.1268 mM) possesses a higher, whereas the oxidized form of 5HDCF-the quinone imine (**1a**_**III**_: EC_50_ 14.37 mg/ml, 0.0463 mM), has a similar toxicity as DCF (EC_50_ 11.36 mg/ml, 0.0357 mM).

Marco-Urrea et al. (2010) also observed a decrease in ecotoxicity (Microtox test with *V. fischeri*) with a concomitant removal of DCF and its transformation products in a culture with *T. versicolor.*

The laccase-mediated transformation of BPA was demonstrated previously by Fukuda et al. (2001). The reaction results in the low-molecular weight product 4-isopropenylphenol and in dimers and oligomers of BPA with a higher molecular weight than BPA (Fukuda et al. 2001; Uchida et al. 2001; Fukuda et al. 2004). Neither product group showed estrogenic activity (Fukuda et al. 2004).

### Transformation of DCF and BPA in secondary effluent

In the tested municipal wastewater, the transformation of DCF and BPA was diminished in comparison with that in phosphate buffer. Although the values for the laccase-mediated transformation with 65% for DCF and 100% for BPA resemble that determined in phosphate buffer, these values were achieved only with 50-fold higher laccase activity than in phosphate buffer. In a mixture of DCF and BPA, the transformation was 2 and 1.5-fold lower, as compared to the individual compounds alone, whereas in phosphate buffer the transformation was higher (in case of DCF) or the same (in case of BPA).

A similar effect was observed by Nair et al. (2013). Thus, the incubation of DCF (in a mixture with BPA and 17-α-ethinylestradiol) in secondary effluent leads to 40% less transformation of DCF. The authors attributed the lower transformation to the presence of organic matter (Nair et al. 2013) which may act as radical scavengers (Ahmed et al. 2012). Nevertheless, the influence of inorganic compounds such as CO_3_^2−^, HCO_3_^−^ or Cl^−^ cannot be ruled out (Ahmed et al. 2012). Thus, chloride and fluoride anions have been described as laccase inhibitors (Xu 1996; Nagai et al. 2002; Ramírez-Cavazos et al. 2014). The effect of such wastewater constituents can be manifold. Beside radical scavenging, the components may influence the structure of the laccase, resulting in insufficient binding of the substrate in the active site, and consequently in less transformation. A reaction of the pollutants with the components is also conceivable preventing binding on the active site of the enzyme. Beyond this the secondary effluent may contain other inhibitors. Thus, azide and cyanide anions as well as l-cysteine have been described as laccase inhibitors (Nagai et al. 2002; Ramírez-Cavazos et al. 2014). Kim and Nicell (2006) described the reduced conversion of BPA in the presence of different anions such as fluoride, sulfide, sulfite and cyanide. The influence of humic acid on the removal of pollutants is also not clear at the moment (Asif et al. 2017a). Finally, it cannot be ruled out that other laccase substrates are present in the secondary effluent, which lower the transformation capacity for DCF and BPA.

The removal of persistent pollutants during wastewater remediation remains a challenge for the future. Efforts to determine the effects of pollutants in a mixture during the water purification process should be enhanced, in particular for enzyme-mediated processes. The decrease of toxicity in the assays containing DCF, BPA or a mixture of both confirms the suitability of laccase-mediated reactions for micropollutant elimination. The enzyme laccase may be an important tool for a greener, and thereby environmentally friendly, remediation process.

## Additional file

**Additional file 1.** Additional tables.

## Abbreviations

ABTS: 2,2′–azino-bis(3-ethylbenzothiazoline-6-sulfonic acid) diammonium salt
AP-ESI: atmospheric-pressure electrospray ionization
DCF: diclofenac
BPA: bisphenol A
FA: flufenamic acid
4′HDCF: 4′-hydroxydiclofenac
5HDCF: 5-hydroxydiclofenac
HPLC: high-pressure liquid chromatography
LC/MS: liquid chromatography/mass spectrometry
WWTP: wastewater treatment plants
