# Supplementary material for: Enhanced laccase-mediated transformation of diclofenac and flufenamic acid in the presence of bisphenol A and testing of an enzymatic membrane reactor
Source: AMB Express. 2018 Feb 24;8:28. doi: 10.1186/s13568-018-0546-y (PMC6890904; doi:10.1186/s13568-018-0546-y)
Supplement: Supplementary file 1 — Additional file 1. Additional tables. [file 13568_2018_546_MOESM1_ESM.pdf]

AMB Express

Supplementary Material

Enhanced laccase-mediated transformation of diclofenac and flufenamic acid in the presence of bisphenol A and testing of an enzymatic membrane reactor

Veronika Hahn[1,2]*, Mareike Meister[1,2], Stephan Hussy[3], Arno Cordes[4], Günther Enderle[3], Akuma Saningong[5], and Frieder Schauer[1]

[1] Institute of Microbiology, Ernst-Moritz-Arndt-University Greifswald, Friedrich-Ludwig-Jahn-Str. 15, 17487, Greifswald, Germany
[2] Leibniz Institute for Plasma Science and Technology (INP Greifswald e.V.), Felix-Hausdorff-Str. 2, 17489 Greifswald, Germany
[3] Atec Automatisierungstechnik GmbH, Emmi-Noether-Str. 6, 89231, Neu-Ulm, Germany
[4] ASA Spezialenzyme GmbH, Am Exer 19 C, 38302, Wolfenbüttel, Germany
[5] EurA Consult AG, Max-Eyth-Str. 2, 73479, Ellwangen, Germany

* Corresponding author: e-mail: veronikahahn@gmx.at, Tel.: +49 3834 554 3872, Fax: +49 3834 554 301

List of contents

Information to Experimental Section **3**

Tab. S1: Structural data of 1a$_{I}$ **4**

Tab. S2: Structural data of 1a$_{II}$ **5**

Tab. S3: Structural data of 1a$_{III}$ **6**

Tab. S4: Structural data of 2 **7**

Tab. S5: Structural data of 1b$_{I}$ **8**

Tab. S6: Structural data of 1b$_{II}$ **9**

Information to Experimental Section

General Methods. The reaction mixtures and isolated products were characterized by mass spectrometry (MS) using electro spray ionization under atmospheric conditions (API-ES) (dry and nebulizer gas: nitrogen; nebulizer pressure: 45 psig; drying gas flow: 10 l/min; drying gas temperature: 350 °C; capillary voltage: 4 kV; fragmentor voltage: 75 V) using an Agilent Series 1200 HPLC system with diode array detector and an Agilent 6120 quadrupole mass spectrometer (Waldbronn, Germany).

For routine analysis, the reaction mixtures were analyzed using an HPLC system LC-10AT VP (Shimadzu, Germany) consisting of a FCV-10AL VP pump, SPD-M10A VP diode array detector, and a SCL-10A VP control unit controlled by Class-VP version 6.12 SP5. Substances were separated on an endcapped, 5-µm, LiChroCART® 125-4 RP18 column (Merck, Darmstadt, Germany) run at a flow rate of 1 ml/min. The solvent system used consisted of methanol (eluent A) and 0.1% phosphoric acid (eluent B), starting from an initial ratio of 10% A and 90% B and reaching 100% methanol within 14 min. Elution with methanol was continued for a further 6 minutes.

Chemicals were purchased from commercial suppliers. All chemicals were used as received.

Structural Data of All Compounds

Tab. S1: 1a$_I$: product resulted from the reaction of **DCF**

**1a$_I$**	**[6-(2,6-Dichlorophenylimino)-3-oxocyclohexa-1,4-dienyl] acetic acid**
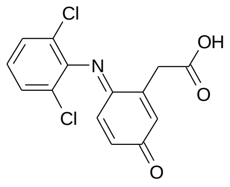	MS-spectrum<br>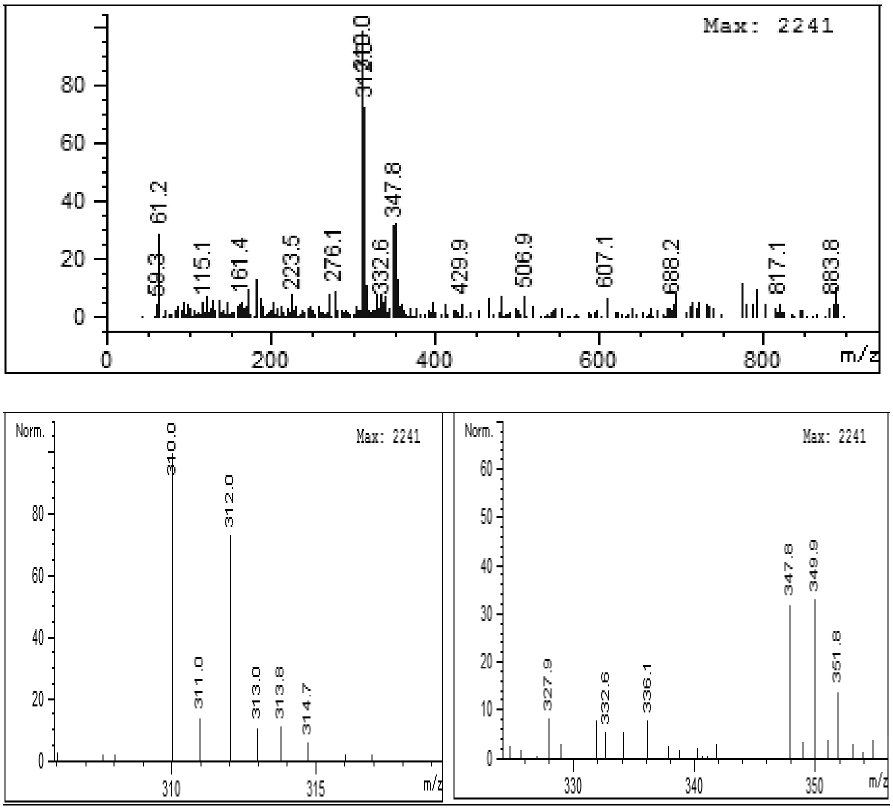

R_f (HPLC) 12.23 min, UV-vis λ_{max} 202, 267, 462 nm. MS *m/z* (rel. intensity) AP-ESI: pos. ion mode $[M+H]^+$ 310.0, 312.0, 313.8 (90, 74, 5), $[M+Na]^+$ 332.0 (9), $[M+K]^+$ 347.8 (32).

Tab. S2: 1a$_{II}$: product resulted from the reaction of **DCF** and **BPA**

**1a$_{II}$**	**[6-(2,6-Dichlorophenylimino)-3-oxocyclohexa-1,4-dienyl] acetic acid**
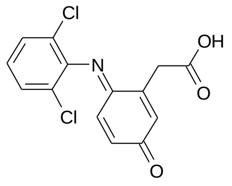	MS-spectrum<br>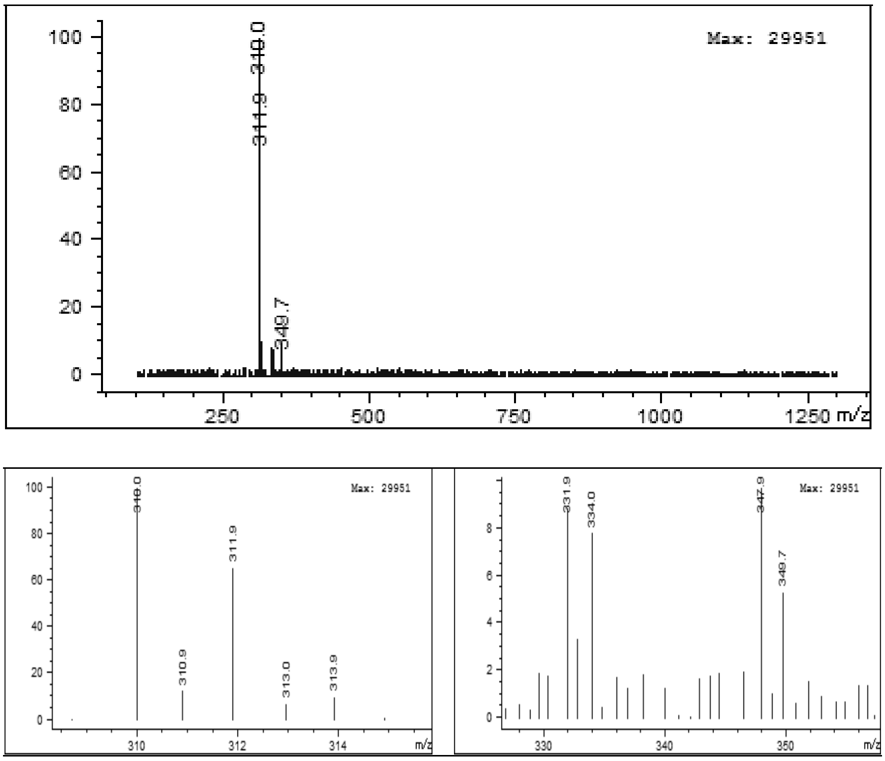

R$_f$ (HPLC) 12.22 min, UV-vis λ_{max} 203, 266, 463 nm. MS *m/z* (rel. intensity) AP-ESI: pos. ion mode $[M+H]^+$ 310.0, 311.9, 313.9 (100, 55, 10), $[M+Na]^+$ 331.9 (9), $[M+K]^+$ 347.9 (5).

Tab. S3: 1aIII: product resulted from the reaction of **5HDCF**

**1aIII**	**[6-(2,6-Dichlorophenylimino)-3-oxocyclohexa-1,4-dienyl] acetic acid**
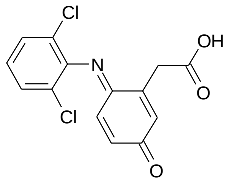	MS-spectrum 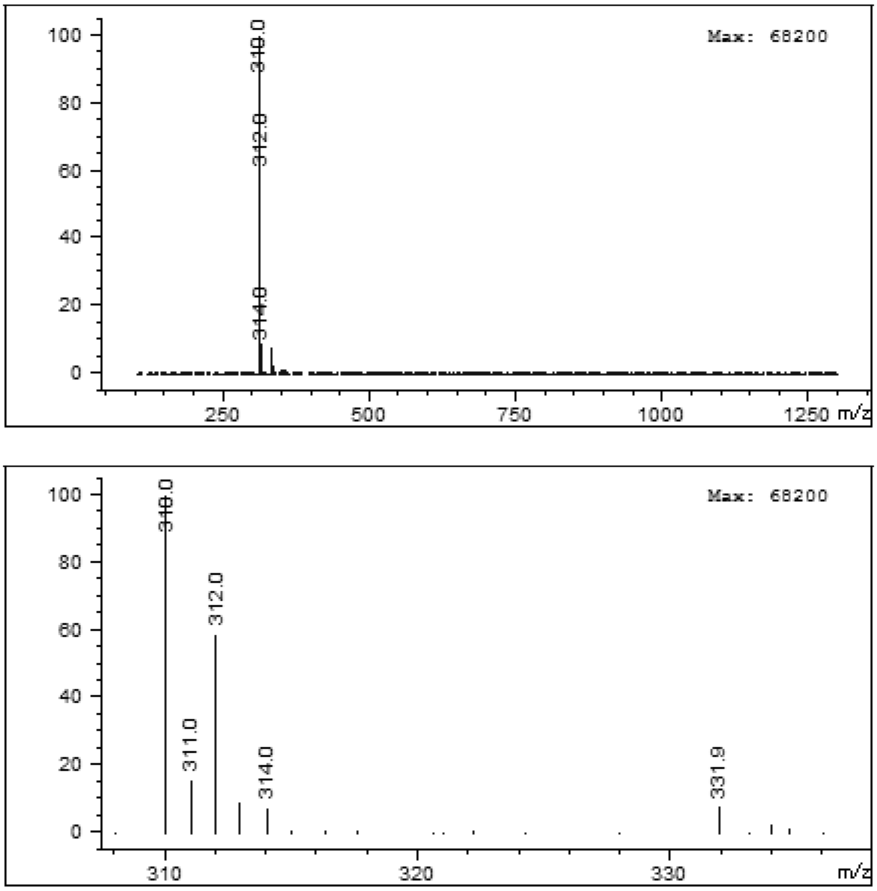

R_f (HPLC) 12.29 min, UV-vis λ_{max} 204, 266, 463 nm. MS *m/z* (rel. intensity) AP-ESI: pos. ion mode $[M+H]^+$ 310.0, 312.0, 314.0 (100, 58, 7), $[M+Na]^+$ 331.9 (6).

Tab. S4: 2: product resulted from **4’HDCF**

**2**	**[2-(2,6-Dichloro-4-oxocyclohexa-2,5-dienylideneamino)-phenyl]-acetic acid**
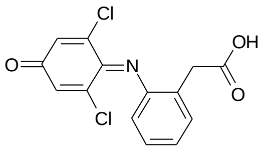	MS-spectrum 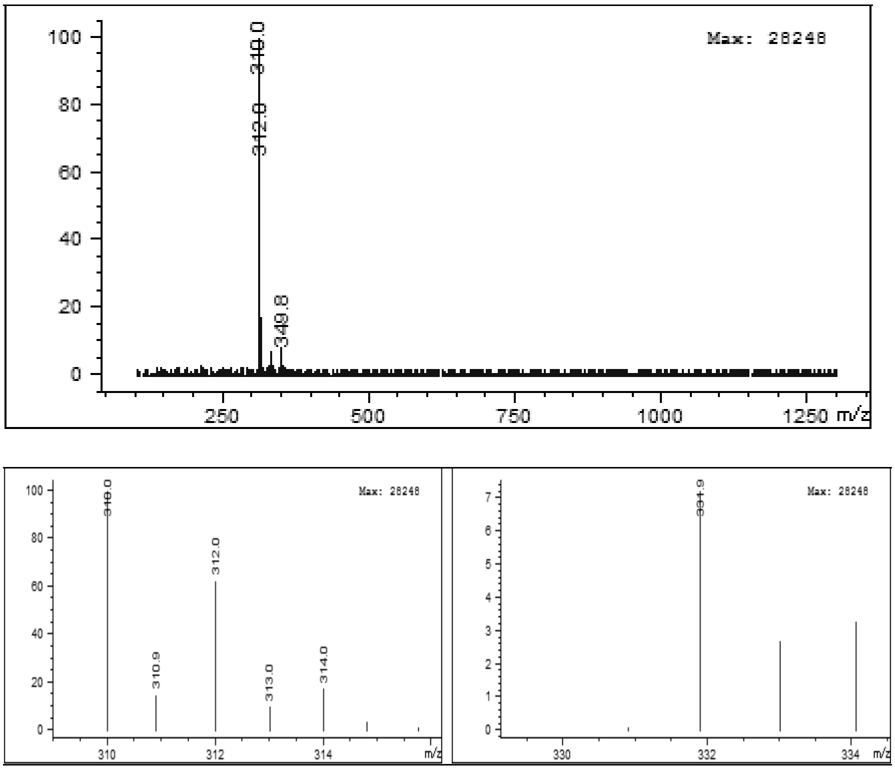

R_f (HPLC) 12.41 min, UV-vis λ_{max} 202, 278, 524 nm. MS *m/z* (rel. intensity) AP-ESI: pos. ion mode $[M+H]^+$ 310.0, 312.0, 314.0 (100, 62, 18), $[M+Na]^+$ 331.9 (7).

Tab. S5: 1bI: product resulted from **FA**

**1b**I	**3-Oxo-6-(3-trifluoromethylphenylimino)-cyclohexa-1,4-dienecarboxylic acid**
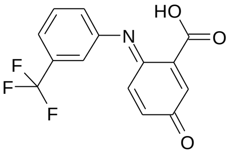	MS-spectra<br>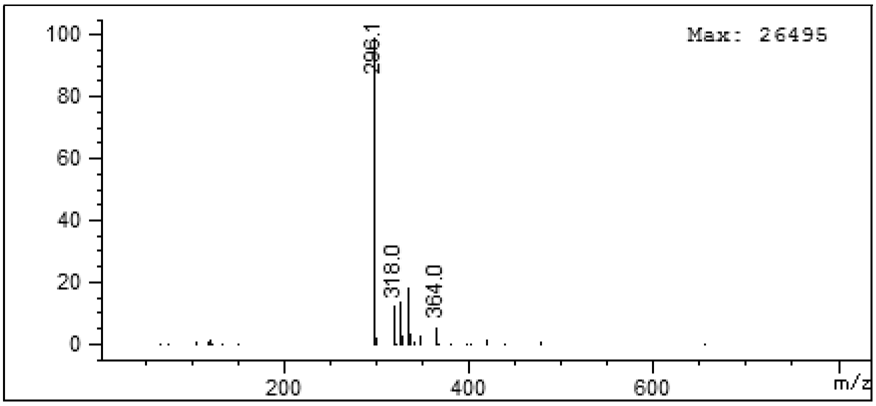<br>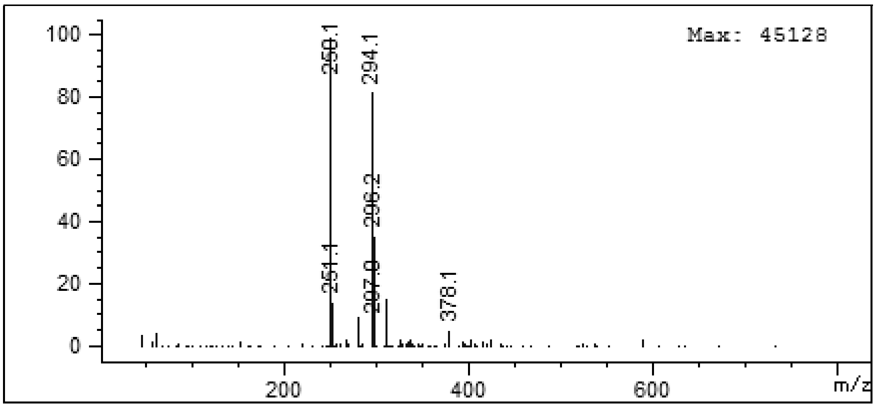

R_f (HPLC) 12.09 min, UV-vis λ_{max} 203, 274, 440 nm. MS *m/z* (rel. intensity) AP-ESI: pos. ion mode $[M+H]^+$ 296.1 (100), $[M+Na]^+$ 318.0 (12), $[M+K]^+$ 334.0 (29), AP-ESI: neg. ion mode $[M-H]^-$ 294.1 (81).

Tab. S6: 1b$_{II}$: product resulted from **FA** and **BPA**

**1b**$_{II}$	**3-Oxo-6-(3-trifluoromethylphenylimino)-cyclohexa-1,4-dienecarboxylic acid**
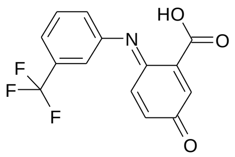	MS-spectra 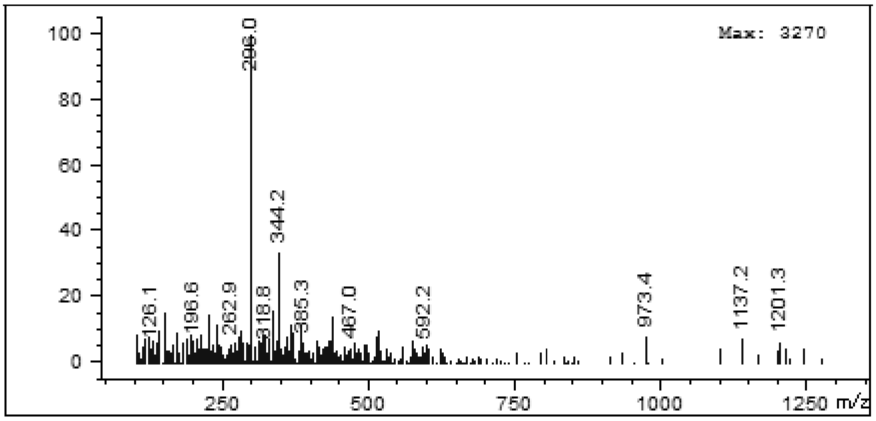 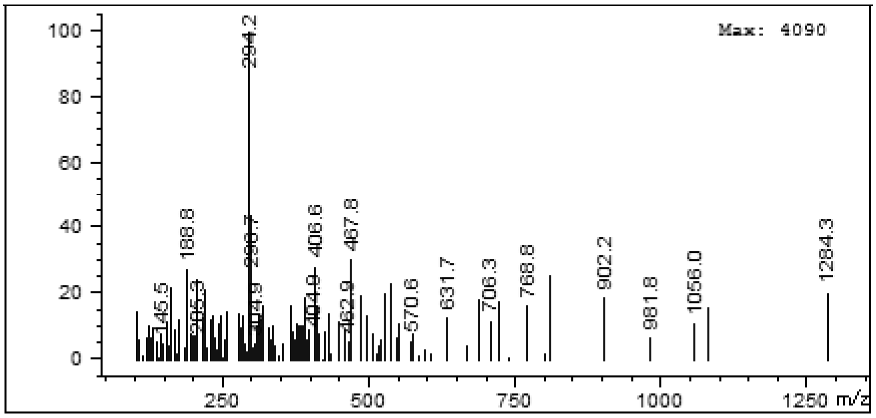

R$_f$ (HPLC) 12.00 min, UV-vis λ_{max} 203, 277, 434 nm. MS *m/z* (rel. intensity) AP-ESI: pos. ion mode $[M+H]^+$ 296.0 (100), AP-ESI: neg. ion mode $[M-H]^-$ 294.2 (100).
